# Solution conformational differences between conventional and CENP-A nucleosomes are accentuated by reversible deformation under high pressure

**DOI:** 10.1063/4.0000857

**Published:** 2025-10-27

**Authors:** Kushol Gupta, Nikolina Sekulic, Praveen Kumar Allu, Nicklas Sapp, Qingqiu Huang, Kathryn Sarachan, Mikkel Christensen, Reidar Lund, Susan Krueger, Joseph E Curtis, Richard E Gillilan, Gregory D Van Duyne, Ben E. Black

**Affiliations:** 1 University of Pennsylvania, Philadelphia, PA, USA; 2 University of Oslo, Oslo, na, Norway; 3 Cornell High Energy Synchrotron Source, Cornell University, Ithaca, NY, USA; 4 Wilson College, Chambersburg, PA, USA; 5 Center for Neutron Research, National Institute of Standards and Technology, Gaithersburg, MD, USA

## Abstract

Solution-based investigation of the physical nature of nucleosomes has its roots in X-ray and neutron scattering experiments, including those that provided the initial observation that DNA wraps around core histones. In this study, we performed a comprehensive small-angle scattering study to compare canonical nucleosomes with variant centromeric nucleosomes harboring the histone variant, CENP-A. We used nucleosome core particles (NCPs) assembled on an artificial positioning sequence (Widom 601) and compared these to those assembled on a natural α- satellite DNA cloned from human centromeres. We establish the native solution properties of octameric H3 and CENP-A NCPs using analytical ultracentrifugation (AUC), small-angle X-ray scattering (SAXS), and contrast variation small-angle neutron scattering (CV-SANS). Using high-pressure SAXS (HP-SAXS), we discovered that both histone identity and DNA sequence have an impact on the stability of octameric nucleosomes in solution under high pressure (300 MPa), with evidence of reversible unwrapping in these experimental conditions. Both canonical nucleosomes harboring conventional histone H3 and their centromeric counterparts harboring CENP-A have a substantial increase in their radius of gyration, but this increase is much less prominent for centromeric nucleosomes. More broadly for chromosome-related research, we note that as HP-SAXS methodologies expand in their utility, we anticipate this will provide a powerful solution-based approach to study nucleosomes and higher- order chromatin complexes.

